# Disparate Emotions as Expressions of Well-Being: Impact of Festival Participation from the Participants’ Subjective View

**DOI:** 10.3390/ijerph20010329

**Published:** 2022-12-26

**Authors:** Saeid Abbasian

**Affiliations:** Department of Informatics and Tourism Studies, Södertörn University, Alfred Nobels Allé 7, 149 81 Huddinge, Sweden; saeid.abbasian@sh.se; Tel.: +46-8-608-51-25

**Keywords:** feelings, well-being, festivals, leisure, immigrants, Sweden

## Abstract

The purpose of this research is that through festival participants’ subjective views get a better understanding of how their participation results in positive feelings and well-being. The paper has a qualitative approach based on a delimited part of a larger survey and the content analysis method has been employed. A total of 280 participants, mainly of immigrant origin, answered one of the open-ended questions in the survey: *What emotions does celebrating the Fire Festival evoke in you? Please justify your answer.* The most common meaning in the answers has been good feelings followed by feelings of integration and community, the return of spring, and nostalgia. The results show that the festival has significance for these peoples’ good feelings and well-being, even though it lasts five hours. This study contributes to increased knowledge of festivals’ positive impacts on individuals, especially on immigrants living in Western countries.

## 1. Introduction

Leisure activities such as participation in cultural festivals are considered to result in a series of positive effects both for people and communities and help create socially sustainable societies [1,2,3]. One of these effects is the individual’s positive feelings after participation. Positive feelings that are created within visitors at a festival are important for the festival’s survival and success [4], decide the visitors’ overall satisfaction, and thereby generate their loyalty to the festival and their willingness to recommend it to other people [5,6]. Nevertheless, it is important to emphasize the significance of positive feelings for the visitors’ well-being, especially when the festival is local and the visitors are mostly locals, repeat participants, and not tourists [7,8,9]. This importance becomes even greater when the visitors are mostly migrants living in the local community. Migrants of the first generation might especially experience difficulties in adaptation to the host society which can negatively impact their psychological well-being [10,11,12,13]. Participation in cultural or ethnic festivals might reduce these feelings among them [1,2].

Many festival studies during recent decades have focused on the economic impact of festivals and regional development (e.g., [14,15,16,17,18]). Other aspects, such as the positive psychosocial and sociocultural effects of the festivals on visitors and communities, have also been touched upon, mostly in quantitative surveys by linking visitors’ emotions to items selected in advance (e.g., [19,20,21]). Relatively few qualitative studies have focused solely on the emotions that have been created through festival participation, and less attention has been paid to cultural festivals arranged by, or for, immigrants, refugees, and ethnic minorities in Western societies. Migrants and refugees have been relatively under-researched groups in recent festival studies [22]. In the other words, there has been a research gap that this paper aims to fill.

Hence, the purpose of this research is that through the Fire Festival (In Swedish Eldfesten) participants’ subjective view, obtain a better understanding of the participation’s positive impact on their feelings and well-being. By subjective perception of well-being at the festival means simply the visitors’/participants’ assessment of good moods and feelings, in their own words, during and after their participation [8]. The paper has the following questions:

Q1. How do the visitors to the Fire Festival describe the feelings that are created by their participation in the festival?
Q2. What can these feelings tell us about their well-being?

In similarity with English, Swedish, and Persian languages, in which the words emotions and feelings are almost synonyms (e.g., [21,23]), here these words are also used as synonyms. Since the Fire Festival visitors mostly come from Greater Stockholm, they are also locals, participants, and attendees at the same time, but they are not tourists. Still, many earlier festival studies that dealt with tourism-related aspects will be touched upon here because of their relevance. This paper is a contribution to the existing body of research on festivals and their impacts on participants’ feelings and well-being, particularly those that concern immigrants and refugees in Western societies.

## 2. Literature Review

### 2.1. Fire Festival in Stockholm

Migration not only impacts the lives of migrants in their new societies but also has an impact on the host societies. The host communities become multicultural and multiethnic; a mosaic of people from different parts of the world bringing their lifestyles, beliefs, cultures, traditions, and experiences to these new societies and enriching the existing culture of the entire society [22,24]. One of these aspects is migrant cultural festivals which are considered a way to achieve faster mutual cultural integration between hosts and migrants [22,24]. An example of such a festival is the non-religious Fire Festival in Stockholm which celebrates the rebirth of nature with fire, that according to ancient tradition, symbolizes among other things health, luck, and prosperity [25].

The ancient Fire Festival is the first part of the annual Nowrouz/Nowruz (Ancient Persian New Year) celebration on March 21. Since 2010, Nowrouz has been recognized by UNESCO as an intangible cultural world heritage [26]. Both Nowrouz and the Fire Festival are celebrated annually in March by several nations in the Middle East, Caucasia, and Central Asia and also by communities from these nations in the Western world. In Stockholm, the festival has been celebrated annually since the early 1980s, mostly by people of Iranian origin in the Stockholm suburbs. The Swedish National Touring Theatre (henceforth Riksteatern) began, in 2010, to also officially organize the festival yearly in downtown Stockholm [25]. Due to the COVID-19 pandemic, the celebration was canceled in 2020 but was restarted digitally (without the public) in 2021 and was broadcasted live through Internet TV. In 2022, it was restarted physically, but due to continued restrictions, fewer members of the public could enter the venue. The festival, which lasts for a maximum of five hours (including the entrance, preparation, and a two-hour live show), consists of live multiethnic music, dance, ethnic food, and the traditional ritual of jumping over fire. The meaning of the festival is to say farewell to winter and welcome spring. The visitors/participants and artists at the festival are mostly of Iranian, Kurdish, Afghan, Tajik, Turkish, and Azeri origin, but there are also a few ethnic Swedes among them. The following research is based on data that were collected after the celebration in March 2019 in which 16,000 people participated [27].

### 2.2. Positive Feelings: Definition and Criteria

Participation in festivals can create a series of positive feelings and emotions for the participants that can all be counted as evidence of well-being and satisfaction with the festival [6]. There are no specific terms in the literature that 100% match the definition of feelings and emotions, and different authors from different disciplines have used different terms and methods to measure, define, and explain feelings/emotions created during a festival. While Grappi and Montanari [19] talk generally about emotional experiences and responses in a festival context, Lee and Chang [28] use, for example, eight emotional experiences in their study; contented, peaceful, optimistic, thrilled, hopeful, encouraged, pleased, and joyful. Positive emotions and feelings at festivals can also be expressed in different dimensions and by using many everyday terms, such as joy, pleasure, happiness, etcetera [29]. Other authors (e.g., [30]) include feelings such as attachment, belonging, unity, and solidarity. One more aspect is the sense of pride as a source of identity-feeling for festival participants [31,32], who in some cases might belong to discriminated groups in the community [33]. Togetherness and a sense of shared experience have been interpreted as other signs of good feelings and satisfaction with the festival [34]. Other authors (e.g., [23,35]) also talk about shared extended emotions that are linked to other people and which differ from personal emotions.

It is not only positive feelings that are discussed in the research into festival participation. Some authors (e.g., [21,28,36,37]) include both negative and positive feelings and experiences such as love, joy, excitement, surprise or anger, sadness, discontent, fear, and also duality/contrasting feelings such as happy–unhappy, disappointed–delighted, annoyed–pleased, bored–entertained at a festival. Additionally, a sense of nostalgia and feelings of being homesick can arise for immigrants in their new countries [38,39], but authors (e.g., [40]) suggest that immigrants’ participation in their own ethnic festivals reduces these feelings. The list of emotions can be much longer if many other positive and negative terms used by other scholars are included.

### 2.3. Relationship between Positive Feelings and Well-Being

A festival, as a typical leisure activity/experience, can ironically be considered as a consumption object and the visitors of the festival as consumers of this object, and thereby all emotions in this context are consumption emotions [37,41], precisely as our feelings are toward the consumption of goods in our materialistic world. Festivals are meeting places for people who share common interests [19,42,43,44] and these meeting places are considered to create happiness for the participants and thereby result in their well-being [45,46,47,48]. The impact of festivals on individuals’ well-being can be studied through the visitor’s own perception of the effects of their participation and happiness is a very immediate sign of well-being through participation [8,49,50,51].

The positive impact of a festival has also been studied with a link to local residents and their emotional encounter and solidarity with tourists visiting the place (e.g., [52,53]), or with a link to social costs and benefits for the residents (e.g., [54]). Yolal et al. [9], who followed the latter track, argued that it is difficult to assess whether locals’ subjective well-being is solely influenced by their participation. With support from earlier research (e.g., [55,56,57,58,59]), Yolal et al., [9] put the issue of social costs and benefits in a broader context including community and educational benefits (e.g., a good image for the community, learning new things) and community costs (e.g., participants’ quality of life, such as the absence of vandalism and increased traffic and community resource utilization). In their theoretical model, they suggest that the locals’ well-being is perceived through the outcome of the included costs and benefits mentioned above, which also determines the sociocultural impact of the festival on them.

Well-being is a term normally used in psychology, and psychosocial effects simply mean positive feelings that are created within the festival visitors through their socialization and interaction with other participants during the celebration and in the long-term through their relationship with the surrounding community [60]. There are two definitions of well-being; subjective well-being refers to, among other things, satisfaction and happiness, and psychological well-being regarding, among other things, meaningful life and personal development [61,62], and Langley and Francis 2016 as cited in [7] and [63]. However, there is a disagreement between scholars on whether positive feelings and well-being are associated with physically or non-physically active participation (e.g., [64]). Positive feelings and well-being can exist both in the short-term [65,66,67] and in the long-term after participation [68,69].

### 2.4. Migrants’ Positive Feelings and Well-Being through Participation

Participation in leisure activities, including festivals, with other people that generally create positive feelings and well-being for the participants [64,70,71,72] can also be applied to immigrants in their host societies [73,74,75]. In other words, good feelings and well-being created through participation in festivals that are universal for all people can also be studied in the context of migration and the aspects linked to it. Hassanli et al. [22,24], for example in their studies in Australia, concluded that immigrants’ and refugees’ participation in multicultural festivals has a positive impact on their well-being caused by their sense of belonging and community. This is confirmed by two other studies in New Zealand [76,77] that emphasize that festival participation not only creates happiness, joy, pride, etcetera but additionally creates a sense of identity, community, and belonging among different generations of immigrants.

In a quantitative comparative study in Germany and the UK, inspired by Bourdieu’s cultural capital theory, Giovanis [75] attempts to link immigrants’ happiness and well-being after participation in sociocultural activities to some hypotheses and variables such as generation, and class (job, income, and education). Both first and second generations of immigrants that have good positions in the host labor market are well-educated, and socioculturally active, and tend to show well-being to much higher extents than other immigrants who lack these preconditions. In this regard, their habits and well-being become more similar to the native’ s, and their cultural capital i.e., language abilities, and knowledge of the host societies’ cultural codes play a particular role.

## 3. Methodology

The following research uses a qualitative approach since the focus here lies on words and not on numbers and figures [78,79]. The research is also based on a delimited part of a larger dataset consisting of 280 complete surveys online. The research team put together a survey with open-ended and closed-ended questions on two homepages in Stockholm throughout April 2019. After the deadline, only 280 completed surveys were accessible and visible while the non-completed surveys were made invisible by the survey tool. In other words, the sample was randomly collected, and the research team had no control over who participated. The 280 respondents, almost entirely from an immigrant background, answered one of the qualitative open-ended questions in the survey that concerned their feelings created through festival participation: *What emotions does celebrating the Fire Festival evoke in you? Please justify your answer*. In other words, answers to this question constitute the dataset for this paper. All these respondents participated in the Fire Festival in March 2019 in Stockholm. They were promised total anonymity in advance and with consideration of the European GDPR (General Data Protection Regulation) law. This means that the author does not know which persons with which personal identities, educational backgrounds, ethnicity, gender, and age, are behind the individual answers and quotations. The language in the questionnaire was Swedish and very few respondents gave their answers in English. The research is inductive, since the author had no theoretical framework in advance, but allowed the data to be analyzed first, and then sought to connect the results to earlier research [80].

**Figure 1:**
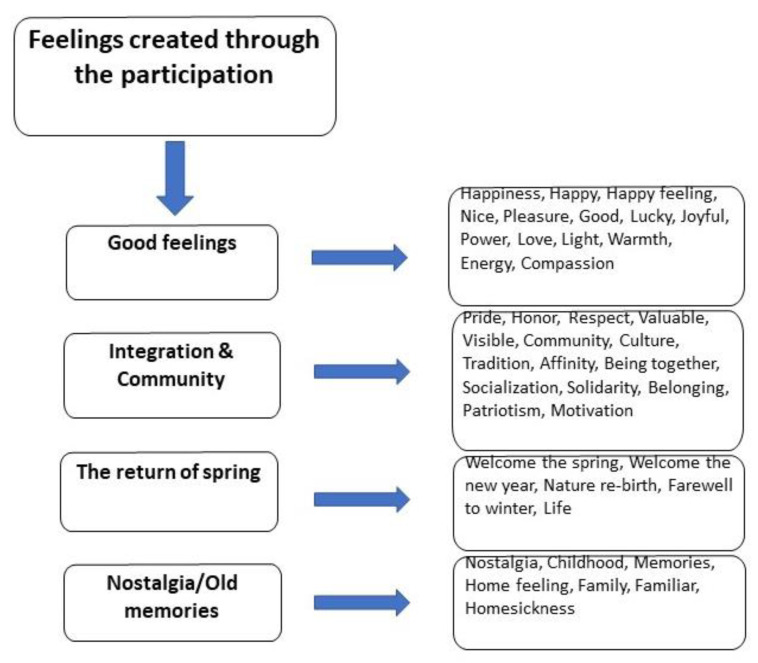
The four categories of meanings in the answers with related codes in each are illustrated by the author.

The content analysis method was assessed to be suitable to achieve the research purpose since content analysis is about the interpretation of a material (e.g., a written document or interview transcript) through the identification of assumptions, themes, patterns, and meanings within it. The analysis is based on codes in the data that all reflect the research question [79,80,81]. Since the approach is inductive (empirically driven), the conventional analysis that refers to coding categories in raw data has been employed [79,82]. The procedure (i.e., reading the main text repeatedly; coding; condensing; discovering layers of meanings, categories, or classifications; linking to theory/earlier research; verifications; conclusions) [79] began with the author moving all 280 answers to the open-set question to a Word document. Then, the answers were reviewed repeatedly to derive a holistic perspective on the document. In the next step, through text coding, visible (manifest) and underlying (latent) meanings were discovered, and four main categories of content (boxes to the left side of Figure 1) were highlighted in connection to the codes in the answers (boxes to the right side of Figure 1) (see Figure 1). In total, 405 words/codes were identified, most of them recurrent in one way or another that addressed feelings and emotions. In the last steps, the author sought to reconnect with relevant earlier research, verified the results, and drew some conclusions. Important results were also highlighted by quotations. The demographic data has been used in this paper only to show the respondents’ backgrounds and is not included in the analysis. In the development of the codes only the author of this paper was involved.

The validation of the codes was based on repetition, induction, and conventional analysis. This means that the author, through repetitive reviews of the answers, included all codes that were relevant to the research purpose and research questions. The author had no controlling codes (ready-made codes) in advance but allowed the data to speak for itself. Through this procedure, manifest content (objective and obviously descriptive codes) was discovered, meanwhile, other codes were discovered through the author’s subjective interpretation of latent content/ underlying the meanings in the answers [82].

## 4. Results and Analysis

The results of the survey’s demographic data in Table 1 show that most of the respondents (64%) were men, and most of them (66%) were older than 40 years of age. Less than half of them had lived in Sweden for more than 21 years and the same for less than 21 years. In the latter, over 16% can be considered newcomers since they had lived in Sweden for less than five years. Only 4% reported that they were born in Sweden. A predominant majority (83%) called themselves Swedish–Iranian, while the rest called themselves Swedish–Afghan, Swedish–Iraqi, Swedish, and others. A large part of them (82%) had an academic education, and 72% were employed in the private or public sector or were self-employed, while the rest were either unemployed, retired, or students. A predominant majority (81%) came from Greater Stockholm and the neighboring municipalities. They came to the festival mostly with family members, friends, and relatives, and most of them were participating in the festival for the second or third time, or more. Most of the respondents had visited the arena (Skansen) previously. Finally, most of them also answered that their expectations were partly or fully fulfilled.

Before starting the analysis, answers from 18 respondents were deemed irrelevant and were removed from the analysis. Of the remaining respondents (262 individuals) many mentioned more than one, up to six feelings, as an answer to the question *“What emotions does celebrating the Fire Festival evoke in you? Please justify your answer”*, and many of them justified their answers with comments. All answers were collected and coded in categories as seen in Figure 1. Here, different from a thematic analysis which touches upon recurrent themes/patterns, there are various categories covering different words/codes that indicate a common meaning. The only recurrent and dominant pattern in the data was the overall pattern of *“good feelings”* that was visible throughout most answers given by the respondents. This was followed by the category of integration and community, followed by the category of the return of spring; the category of nostalgia/old memories also appeared frequently in the answers. It was noted that very few answers were linked to the category of bad feelings, however, they were also included in the analysis.

### 4.1. Good Feelings

In this response category, the words happy, happiness, and happy feelings were most frequently used by many respondents which can be signs of good feelings. A critical reflection here would be to wonder if these happy feelings were purely the outcome of the celebration of an ancient tradition regardless of the organizer, or if they were the results of the whole context, i.e., socialization with others in dancing and enjoying the entertainment. The following quotations are representative:

*I Feel happy to celebrate the tradition of our ancestors.*

*I get happy when I am with people from my country.*

*One becomes happy that there is such an opportunity to be able to celebrate the Fire Festival with a lot of other people and to be able to pass on this tradition to the children who can experience it in the best possible way.*

This result is partly in line with earlier research (e.g., [21,29]) which emphasized that the most important outcome of the festival participation for the participants is happy feelings.

Additionally, in this category, codes/words such as nice, good, strong feelings, many feelings, pleasure, lucky, joy, joyful, lovely, hope, warmth, energy, power, light, love, humanity, compassion, kindness, peace, harmony, calm, free, exciting, wonderful, fantastic, satisfied, and curiosity were used. These codes/words bear no negativity, convey positive feelings, and can be interpreted as good feelings. They can address different things, above all, the positive mood that is created in the festival through celebrating with other people. The result is consistent with some of the earlier research (e.g., [28,37]). The following quotations are also illustrative and representative:

*Very, very nice feelings. I was extra happy when I saw so many Swedes were there and celebrated with us.*

One of the respondents referred to her/his answer to an earlier question, question 11, that concerned their motivations behind participation:

*Explained in Q11, but as I mentioned, mostly the feeling of joy and belonging to a society.*

*It feels good when you see the people are happy and dancing.*

### 4.2. Integration and Community

This category contained the words pride, honor, respect, valuable, visible, community, affinity, belonging, being together, socialization, solidarity, identity, culture, tradition, patriotism, and motivation, which can be signs of positive feelings. The use of these words, however, mainly expresses a desire for the integration of the ethnic community, including belonging, community, and inclusion in society, and is against invisibility, ethnic segregation, exclusion, and isolation [22,24,76,77]. The fact that such a cultural festival is organized by the Swedish Riksteatern indicates recognition of the tradition at the national level, something that can be important for some minority groups that might have experienced feelings of discrimination [33]. This creates pride, honor, respect, visibility, and identity for these people who see their ethnic communities and cultural traditions being noticed by mainstream society. This result is in line with some of the earlier research (e.g., [30,31,32,34]). Some illustrative quotations are:

*A certain feeling of togetherness, the joy of gathering and celebrating together, and that the tradition is noticed and returns every year.*

*The feast itself reminds me of a long history we have had in Iran. The Fire Festival in Stockholm is an identity-creating phenomenon for Iranians who live here. Apart from differences and sometimes conflicts that exist among them…*

*When you see how big and organized it is, you feel pride and belonging and community with everyone else who likes to celebrate with me.*

### 4.3. Nostalgia/Old Memories

This category includes the codes/words nostalgia, childhood, memories, home feeling, family, familiar, and homesickness. Nostalgia and old memories are generally feelings that might be difficult to place in terms of certain specific positive or negative feelings since they are about missing the past years of one’s childhood or youth. However, homesickness or missing family members are sad feelings that arise among immigrants in host societies (e.g., [38,39,40]), especially during their first years and before they become more adapted to the new society. Perhaps a critical reflection here is that for a part of these people “home” is still far away, somewhere in the Middle East and not yet in Sweden. Is it a sign of lacking integration in society and thereby still feeling like a stranger here?

*Homesickness, and missing Iran, remind me of my childhood memories.*

*Reminds me of good old times in the homeland when everyone lived in peace.*

*As I said, my great memories from childhood and the feeling of togetherness and that I and my culture as part of this society are valuable and seen.*

### 4.4. The Return of Spring

This category includes the words/codes welcome the spring, welcome the new year, nature re-emergence, farewell to winter, and life, which can all be associated with positive feelings. The respondents address mother nature’s rebirth after a long and dark autumn and winter and the arrival of the spring season, which is symbolically associated with new life and brighter days. This feeling of new life is quite reasonable for all participants since a few days after this feast, the spring season begins both in Sweden and in the Middle East and the days become brighter and longer, and if not yet in Sweden, in the Middle East this transition is evident in nature with emerging greenery. In Iran and Afghanistan, the calendars also turn into a new solar year on 21 March.

*Childhood memories in the first place... but also that life begins again in the spring.*

*That I look forward to the new year and the spring that will appear when the days will be longer, buds will become flowers, etc.*

### 4.5. Bad Feelings

This category includes few answers but several codes and words such as nothing, no happy feelings, loneliness, anger, upset, and dissatisfaction. The first three words can be associated with the person’s own life, while the latter words are more associated with critical views about the organization of the festival by Riksteatern. Although few codes are included here, they are still partially consistent with some earlier studies (e.g., [28,36]). Below is a quotation from one who was dissatisfied and angry with the content of the festival which according to him/her lacks originality and authenticity:

*I become angry. Everything has changed. Next year you can have Santa Claus at the Easter celebration, so you understand what that means.*

## 5. Discussion

The purpose of this research has been to get a better understanding of the Fire Festival participants’ subjective view on the impact of this festival on their feelings and through them, their well-being. This study differs from many other studies with a quantitative approach. The respondents in this study had the opportunity to express their feelings in a large number of disparate words and through comments and not by ticking off a list of preset answers as suggested in some other large quantitative surveys. This festival has been a meeting place for several ethnic groups in Stockholm, groups who share a common interest [19,42,43,44].

The data support the existence of a link between participation, positive feelings, and well-being, and when put into the context of earlier research, it confirms previous findings. The respondents, in unison, have expressed that almost all positive feelings such as happiness were created through their participation in the festival. These feelings are consistent with earlier research that found a relationship between disparate aspects/criteria of positive feelings such as happiness [6,29,45,46,47,48], inclusion, belonging and integration [22,24,30,31,32,33,77], nostalgia [38,39,40] and their common positive impact on the participants’ well-being. Since the most frequently expressed feeling in this research has been happiness, the results also support earlier research [49,50] that claimed that a very immediate sign of participants’ well-being is expressed happiness. These positive feelings can also be an indicator of satisfaction with the festival to a large extent [4,6,68]. Since many of the respondents talked about feelings and emotions that were linked to integration and inclusion at the macro/community level, it can also be a sign of shared extended emotions [23,35] with other participating ethnic groups, i.e., to see that the ancient ancestors’ legacy has been maintained and recognized by the host society.

The results of this research give an opportunity to somehow reflect on Giovani’s [75] research or rather draw an assumption linked to it, although demographic data has not been analyzed in this paper. Although we did not ask any questions relating to generational status, it can be seen in the data that both the first and second generations of immigrants and also newcomers have been among the respondents and participants. A predominant majority of the respondents have been people with capital resources such as jobs, income, high education, and surely, through their jobs, access to influential social networks and knowledge of the host society’s cultural values and codes. Could these factors have contributed to their satisfaction, happiness, and well-being? [75]. Perhaps these successful persons also experience psychological well-being from, among other things, a meaningful life and personal development [61,62] (Langley and Francis 2016 as cited in [7,63]) that might not be possible for many other individuals who participated in the same festival to achieve. All these issues need to be further investigated in new research with new questions. Another question to raise is whether these feelings of well-being can be long-lived [68,69] or are only short-lived [65,66,67].

Regardless of the socioeconomic backgrounds of the participants and their degree of adaptation to society, Riksteatern’s arrangement of such festivals gives these individuals hours of happiness and good fortune at the end of a dark Swedish winter, and it is recommended to be continued. It is especially important for newly arrived immigrants and refugees that might feel an identity vacuum. Hopefully, these good feelings give them a sense of ethnic identity and create more positive attitudes toward the new society, and speed up their process of adaptation.

One shortcoming of this research is that, on one hand, a predominant majority of persons of Iranian origin answered the question, and on the other hand, they are also individuals who are well-educated and well-integrated into society. Therefore, it is suggested that these results are taken with caution. This study was not able to include people with different socioeconomic backgrounds, and one reason for this might have been the use of the Swedish language in the questionnaire. This factor may have excluded additional respondents, especially many newcomers who still have difficulty expressing themselves in the Swedish language. A questionnaire in Persian and Kurdish would most likely attract many more individuals and facilitate their ability to answer the questions. This survey could also be completed with focus group interviews or individual interviews to gain deeper insights into the issues.

## 6. Conclusions

The results of this paper show how participation in cultural festivals can create disparate but overall positive feelings among participants (here, immigrants). These feelings, as confirmed by earlier research, can also result in the short-term or long-term well-being of the participants. By creating positive feelings in immigrants, such festivals can accelerate immigrants’ integration into their new societies and contribute to creating more socioculturally sustainable societies. Therefore, the author concludes that this research has implications for Riksteatern as well as for policymakers at a national level and regional authorities in Stockholm county. Further, the author concludes that it is of relevance to create festivals that give platforms for more in-depth social and cultural interactions between participants of different ethnic groups and native participants. This kind of festival certainly has even more positive impacts on both participants and the local community. It is difficult to believe that the Fire Festival that lasts for a maximum of five hours can create such a platform. Finally, this research contributes to the existing but rather limited research on immigrant festivals in Western societies and their importance for immigrants’ sociocultural integration into these societies.

## Figures and Tables

**Table 1:** Sociodemographic data of the respondents.

Gender	64% Men, 36% Women
Age	66% were aged more than 40 years old
Years in Sweden	About half had lived in Sweden for less than 21 years, 4% were born and raised there, and the rest had lived here for more than 21 years.
Nationality/ethnicity	Swedish-Iranian (83%), Swedish Afghan (6%), Swedish Iraqi (1%), Swedish (4%) and other (6%).
Educational level	82% had an academic education. The rest had elementary school and high school education.
Profession	72% were employed, 13% were students, and 15% were unemployed or retired.
Residential area	More than 81% of them came from Greater Stockholm. The remainder came from counties in immediate proximity.
Lone participant or in company?	17% came alone while 83% came with friends, relatives and family members.
Participation in the Fire Festival in Stockholm in previous years	62% had been participating for 3 years or more, 18% participated for second time and the rest participated for first time.
Previous visits of Skansen	More than 73% had visited Skansen prior to the festival
Are your expectations fulfilled?	54% believed that the festival had fully fulfilled their expectations, 25% believed that their expectations were partly fulfilled, 21% believed that their expectations were not fulfilled.
